# Everyday social contexts influence fluctuations into and out of chronic pain: an ethnographic study in England

**DOI:** 10.1136/bmjopen-2025-111270

**Published:** 2026-04-01

**Authors:** Samantha Stone, Anica Zeyen, Rachael Gooberman-Hill

**Affiliations:** 1 Bristol Medical School, University of Bristol Faculty of Health Sciences, Bristol, England, UK; 2 School of Business and Management, Royal Holloway University of London, Egham, England, UK

**Keywords:** Chronic Pain, QUALITATIVE RESEARCH, Family, Health, Longitudinal studies, Social Support

## Abstract

**Abstract:**

**Objectives:**

The research explored how individuals experience chronic pain within their everyday social contexts over a 12-month period. The study focused on the interplay between pain and social worlds, through analysis of experiences of social relationships included in engagement in meaningful activities such as hobbies and work.

**Design:**

Drawing on ethnographic approaches from social science, the study involved 295 research visits with 19 participants living with chronic pain (totalling approximately 418 hours of fieldwork) and 48 semistructured interviews (around 30 hours).

**Setting:**

The study was carried out in South West England, UK.

**Participants:**

19 participants were identified through the Avon Longitudinal Study of Parents, 12 women and 7 men, all identifying as white British, aged between 32 and 33 years.

**Results:**

The analysis identified three key themes: (1) Social connections and everyday fluctuations in chronic pain; and (2) the interplay between work, family roles and fluctuations in chronic pain; and (3) Social spiralling and fluctuations in chronic pain. The third theme builds on and combines the patterns identified in the first two themes, drawing together how changes in social connections and balancing of roles coalesce in the experience of fluctuation in chronic pain. Relationships, roles and how these were experienced varied across participants, but all of their descriptions indicated that the constant flux was understood, even if financial or other circumstances meant that people were unable to exert agency that would have proved beneficial. Across themes, interconnected social processes appear to shift and move together, amplifying their collective impact on the experience of chronic pain.

**Conclusion:**

Fluctuations in chronic pain were complex, shaped by and entangled with social contexts that vary in meaningful ways. The findings suggest that to address chronic pain effectively, health and social care may need to move beyond individual-level solutions to consider the multiple, interacting layers of influence that shape and sustain the experience of chronic pain.

**STRENGTHS AND LIMITATIONS OF THIS STUDY**

- A strength of this study was the longitudinal, ethnographic data collection that provided understanding of how social dynamics and the experience of living with chronic pain changed over time.
- The research was co-designed with public contributors who also inputted as the research was carried out.
- All study participants identified as white British, underscoring the need for future research to include greater ethnic diversity.
- The study generated detailed, in-depth insights into social processes, norms and relationships that shape the experience of living with chronic pain, offering analytically robust understandings that may meaningfully resonate with and inform, other groups and populations.

## Introduction

 Chronic pain is persistent or intermittent pain that occurs in one or more places in the body and lasts for longer than 3 months.^1^,^3^ Chronic pain is a global health issue affecting people across all age groups, with just under 28 million, representing approximately 43% of the population reported to be living with chronic pain in the UK today.^1^,^3^ It poses complex, evolving challenges influenced by biological, psychological and social factors. Corbin and Strauss^4^ illness trajectory framework outlines phases of chronic illness shaped by both medical conditions and responses from patients, families and healthcare professionals. While a useful way for identifying shared and unique stages (including pretrajectory, onset, crisis, acute, stable, unstable, downward and dying) to an extent, the framework could be interpreted as implying a relatively predictable, sequential progression.^5^ This may not reflect the unpredictable, fluctuating nature of people’s experiences of living with chronic pain.^6^ In contrast, Von Korff and Miglioretti^7^ describe chronic pain as a dynamic condition, better understood as fluctuating over time with transitions between varying levels of severity, rather than a static experience. Eccleston and colleagues^8^ expand on this with a framework of pain transitions in which individuals move between different states or experiences of pain. For example, transitions may be from acute (short-term) to chronic (long-term), from pain to recovery or between varying levels of pain intensity (high or low impact). While social factors are known to influence individual differences in pain intensity, pain interference, coping capacity and chronic pain meaning-making, the mechanisms underlying these variations and the role of social contexts are not well understood.^8 9^

Recent research has increasingly focused on how individuals live with chronic pain, particularly how pain shapes everyday routines and relationships. Qualitative studies show that as pain intensifies, people in pain often rely more on family and friends for support with self-care and everyday tasks,^10^,^12^ such as household chores^13^,^20^ or shopping.^10 15 21 22^ Families adapt activities with varying success.^1723^,^25^ Chronic pain can also disrupt previously shared meaningful experiences, leading some individuals to withdraw from social settings to avoid stigma.^2325^,^29^ These challenges affect not only physical functioning but also personal identity. Exploring identity, self-concept and social roles offers insight into how people navigate the complexities of chronic pain in everyday life.^30 31^

The socioecological model, rooted in Bronfenbrenner’s work on how individuals engage with and are shaped by their surrounding environment,^32 33^ has been widely adapted across disciplines. McLeroy and colleagues^34^ extended the model to health-related behaviour change, identifying five levels of influence: intrapersonal, interpersonal, institutional, community and public policy. This multilevel framework highlights the need for coordinated change across interconnected levels; a principle now central to public health discourse.^35^ In pain research, Kapos and colleagues^36^ applied socioecological, intersectional and life course perspectives to examine how pain is shaped by interpersonal dynamics, community group structures and broader societal systems. The authors highlight how multilevel social factors differentially shape pain experiences and accumulate over time, including both individual and intergenerational patterns of risk. With a similar focus on the social aspects of pain, Rysewyk and colleagues^37^ conducted a review and identified eight key themes that characterise the lived experience of chronic non-cancer pain, highlighting its emotional, social and identity-related disruptions, and calling for a more empathetic, multidimensional response in healthcare and workplace policy. A recent systematic review of qualitative studies explored how social phenomena intersect with the experience of transitions into and out of chronic pain, underscoring the need for longitudinal, prospective research to understand these processes over time.^38^

Together, existing reviews and their constituent studies indicate the importance of a more socially informed understanding of pain, which is needed to underpin more effective healthcare. Previous studies have begun to characterise the social influences on pain, but they also demonstrate a need for more longitudinal work to understand transitions to and from chronic pain. A clearer understanding of these fluctuations may help identify when and how to intervene to prevent the development of chronicity or reduce the impact of chronic pain on everyday life and work. To address this, our study examined pain fluctuations among individuals followed over a 12-month period.

## Methods

To examine the complexity and interconnectedness of living with chronic pain, this study used ethnographic methods, which is an established field-based approach that combines observation and interviewing to understand how people make sense of their experiences within their sociocultural context.^39^ Ethnography was selected because the study sought to examine not only what participants experienced, but how and why those experiences unfold over time, across different situations and social settings. Ethnographic knowledge is generated through sustained engagement, enabling meaning to be revisited, questioned and refined as relationships develop and understanding deepens. The study is reported in keeping with the Standards for Reporting Qualitative Research.^40^

### Participants and recruitment

The study was conducted in South West England, with participants identified through Avon Longitudinal Study of Parents and Children (ALSPAC) cohort.^41^ ALSPAC is a birth cohort study that recruited pregnant women with expected dates of delivery between 1 April 1991 and 31 December 1992. The subsequent children, parents and families have been followed up ever since, aiming to understand influences on health and development across the life course.^42 43^ The study website contains details of all the data that is available through a fully searchable data dictionary and variable search tool.^44^

A purposive sampling strategy was used to identify adults with chronic pain lasting 3 months or more. When participants were aged approximately 30 years, based on responses to chronic pain questions in the Aches and Pains Questionnaire administered to the ALSPAC cohort.^45^ These questions were: ‘Have you had any aches or pains that have lasted for a day or longer within the past month?’ and ‘If yes, when did the pain start?’. Potential responses to the question about when the pain started were: ‘Less than 3 months ago’, or ‘More than 3 months ago’. Participants in ALSPAC were eligible to take part if they had any form of chronic pain that had started more than 3 months ago, which therefore included both chronic primary pain and chronic secondary pain. Potential participants were not eligible to take part if they had a diagnosis of a terminal condition that meant that they may be near the end of life, to reduce any potential burden on those individuals. Of the 942 individuals identified, 888 lived within 25 miles of the host university and were selected to enable local data collection.

Using a phased approach, we aimed to recruit 20 participants to ensure depth and diversity, in line with qualitative research standards and likely achievement of information power.^46^ Study invitations and information booklets were sent to 300 participants (50 per month, over 6 months), resulting in 30 expressions of interest. After 30 follow-up calls, 19 provided their written informed consent to take part and 11 declined or were unavailable due to work and family commitments. The sample included 12 women and 7 men, all identifying as white British, aged 32–33 years and living in South West England. One participant withdrew after the first meeting for personal reasons unrelated to the study. We also included 10 family members, friends and support workers to help contextualise participants’ experiences. While their input informed the broader study, their contributions are not analysed here. Pseudonyms are used throughout to protect confidentiality.

#### Patient and public involvement statement

A public contribution group of seven people living with chronic pain met quarterly with the research team to inform research design, conduct and interpretation of findings.^47 48^ The group met 10 times between September 2022 and May 2025 (September 2022; January 2023; May 2023; September 2023; November 2023; April 2024; July 2024; October 2024; January 2025; May 2025). Their input shaped recruitment materials, guided emerging areas of inquiry and informed our understanding of how developing themes resonated with their experiences of chronic pain. This collaborative approach was grounded in shared decision making and partnership.

#### Data collection

Between July 2023 and February 2025, the researcher (SS) conducted 295 ethnographic visits over a 12-month period, meeting participants approximately twice per month and spending around 418 hours in total with them and their close social circles. Visits typically lasted between 1 and 2 hours, although duration varied considerably, ranging from 20 to 330 min.

Data collection drew on a range of ethnographic methods, including participant observation, informal conversations, semistructured interviews and the use of visual materials. The fieldwork process was deliberately flexible, with the timings, frequency and location of visits shaped by participants’. A reflexivity journal was maintained throughout to critically examine how the researcher’s attributes, background and assumptions may have influenced interactions with participants and the interpretation of data.

Through sustained observation and ongoing interaction, ethnographic engagement enabled examination of how experiences of chronic pain were shaped by social relationships, normative expectations and broader structural conditions. Observational encounters and informal conversations were treated as analytical sites through which everyday practices and social dynamics surrounding chronic pain could be interpreted and questioned. Field notes were written systematically during or immediately after each encounter to capture both descriptive detail and reflexive insight. Analytical attention encompassed verbal and non-verbal forms of communication across a range of settings, including participants’ homes and public spaces such as cafes, supermarkets and local green spaces.^39 49^ Iterative movement between observation, informal interaction and field conversations informed the focus and conduct of subsequent interviews, while recorded semistructured interview discussions, in turn, shaped ongoing observational attention. As the researcher became increasingly embedded within participants’ routines and social worlds, new insights emerged over time, supporting a responsive and participant-led understanding of experiences in fluctuations to and from chronic pain.^50^

48 semistructured interviews were conducted during research visits, ranging from 13 to 68 min in length (average 40 mins), with audio-recorded informed consent. Interviews typically took place several months into fieldwork and explored topics such as daily life, work and social connections. Topic guides were informed by ongoing analytical engagement during data collection, input from public contributors and existing literature (see online supplemental file 1: topic guides). This flexible and iterative approach enabled in-depth, participant-led inquiry that extended beyond what is typically possible through one-off interviews.

Visual materials, including participant-generated images, metaphors and timelines, were used to facilitate dialogue and reflection on social life and experiences of chronic pain. While not directly analysed here, these materials shaped participant reflections during fieldwork and semistructured interviews. A detailed analysis of the visual data will be reported separately.

#### Analysis

Ethnographic analysis is an iterative and reflexive process that unfolds throughout the research, rather than being confined to a discrete post-fieldwork phase. Drawing on Davies’^49^ reflexive approach, analysis was treated as a continuous practice that began in the field and extended beyond it.^49^ As Davies argues, ethnographic analysis is inherently embedded in the process of data generation, as researchers are continually interpreting, selecting and giving meaning to social interactions as they occur. The act of observing, deciding what to record and determining how events, interactions and experiences are represented already involves analytical judgement shaped by the researcher’s positionality, assumptions and theoretical sensitivities. Reflexive attention was therefore paid to how interpretations were formed in situ, including moments of uncertainty, surprise, tension and discomfort, which were used to deepen understandings of social relations and the researcher’s own role within them. Following fieldwork, analytical engagement continued in a more systematic and sustained manner through repeated critical engagement with fieldnotes, interview transcripts and reflexive writings.

Thematic analysis, which is widely used in UK health research, was guided by Braun and Clarke’s reflexive approach^51 52^ and complemented by ethnographic techniques to organise and identify patterns across the data.^53^ Reflexive thematic analysis was used to examine patterns of meaning across interviews and field notes, understood collectively as a single, integrated data set. Analytical work involved iterative cycles of familiarisation with fieldnotes and interview transcripts, followed by line-by-line coding that remained attentive to context, relational dynamics and reflexive insights generated during fieldwork. Coding was treated as an interpretive and provisional process, with codes being continually refined, combined or discarded as analytical engagement deepened. A coding log was maintained to document analytical decisions, emerging questions and shifts in interpretation over time.

Although thematic analysis is sometimes criticised for breaking complex interconnected information into disconnected parts, the reflexive approach articulated by Braun and Clarke aligns closely with ethnographic commitments to context, reflexivity and meaning making. Therefore, field notes and the interview transcripts were analysed together, rather than as separate data sources, enabling the development of overarching thematic areas that remained grounded in the context in which data were produced. In the results section, illustrative examples are drawn from both field notes of observations and transcripts of interviews, comprising 10 excerpts from semistructured interviews and 11 excerpts from field notes. This balance is intended to demonstrate that analytical interpretations were developed across all data sources. Taken together, reflexive ethnographic analysis shaped how meanings were initially interpreted and questioned in the field, while reflexive thematic analysis provided a flexible yet rigorous framework for developing and articulating meaning across the rich dataset.

Interviews were transcribed, anonymised and checked for accuracy. SS led the analysis using NVivo Software,^54 55^ engaging in iterative review of the dataset to identify patterns linking social phenomena to fluctuations in chronic pain. To support analytical reflexivity and rigour, RG-H reviewed and discussed coding across a sample of 20% of transcripts and fieldnotes, selected randomly across participants and time points.

Three overarching thematic areas were developed (1) social connections and everyday fluctuations in chronic pain, (2) the interplay between work, family roles and fluctuations in chronic pain and (3) social spiralling and fluctuations in chronic pain. The third theme builds on and combines the patterns identified in the first two themes, drawing together how changes in social connections and balancing of roles coalesce in the experience of fluctuation in chronic pain. While these themes were prominent, we acknowledge that other aspects also play a role, and we do not claim the analysis is exhaustive or that it captures all experiences.

## Study findings

Participants described a wide range of chronic pain experiences, varying in location, type or origin and duration (table 1). These included musculoskeletal pain, neuropathic pain and pain linked to long-term health conditions. The duration of pain ranged from childhood onset to several years, highlighting its persistent and fluctuating nature. Despite these differences, participants offered rich and varied accounts of how pain affected their everyday lives. From a constant, all-consuming dominating presence to intermittent episodes and background presence. Participants reflected on complex social dynamics, encompassing relationships, activities and work that intersected with and influenced their experiences of living with chronic pain. While some found routine and connection helpful for maintaining normalcy, others felt that these same demands intensified the challenges of managing the pain.

**Table 1:** Participant demographics (N=19)

Characteristics descriptives		
Gender	Man	7
	Woman	12
Age group	All	Range 32–33 years
Pain duration	All	Range 5–28 years
Pain types include	Body location: back, shoulders, head, jaw, leg, knees, ankle, feet. Reported diagnosis: endometriosis, adenomyosis, polycystic myositis, chronic recurrent multifocal osteomyelitis, functional neurological disorder, cavernoma, cysts, arthritis; fibromyalgia; myalgic encephalomyelitis.	
Employment types include	Self-employed; consultancy; full-time; part-time; compressed hours; shift work; zero hour; unemployed; volunteer.	
Living circumstances	Living with parents	<5
	Living with partner	11
	Living with children	8
	Living alone	<5
Participants with children under the age of 18		10

### Social connections and everyday fluctuations in chronic pain

Participants highlighted the importance of feeling connected, understood and receiving meaningful support in helping them to navigate life with chronic pain. The composition of social networks varied but typically included a mix of close personal relations, family and friends, work and hobby-related connections, as well as more casual acquaintances within the local community. Participants valued the quality of these relationships defined by depth, trust and emotional support, over the number of social connections. Several participants identified humour as one of their most important resources for managing chronic pain, offering distraction and moments of relief amidst their constant pain (table 2, quotation 1).

**Table 2:** Social connections and everyday fluctuations in chronic pain

Quotations	
1.	It almost tricks you into thinking something’s funny, therefore you’re not in that much pain or it can’t be that bad because you’re laughing. You’re making a joke, so it can’t be that bad. It is that bad, but you’re trying to, take the heat off (Kayla, semistructured interview)
2.	Yeah, they’d say they understand but when it suits them, they’ll conveniently forget about [the pain], because they want something. Or they’ll say like oh I’m going to leave you alone today, so you think oh great. But then they’ll ring me for any minor insignificant thing. Or they do the ‘Oh I know you’ve got pain, but I thought you’d want to know this’ or ‘If you didn’t have pain you would want to do this’ … so in other words I want you to do this but I’m going to make you feel bad about it now so that you will do it (Kayla, semistructured interview)
3.	The pain in his back, shoulder and knees has been getting worse over the last few weeks. The last 3–4 days he has avoided social situations - yesterday his brother and sister invited him for breakfast, but he said no. Yesterday he stayed home alone all day. We talked at length about his partner, as they have been finding it difficult to get along lately. Gabriel’s partner is stressed about money for the holidays (Gabriel, field notes)
4.	It’s a mixture because yes, the [dog] is a good companion and requires a lot of love and attention which is a good distraction. However, he also can make my pain worse because I have to take him for a walk and that can be a lot of effort and the pain in the chair going over the bumps, and also having to pick up the poop from the chair and bending down, it hurts my back (Jessica, semistructured interview)
5.	The cats help me to find the joy, I think it’s really important because if you just live in the pain, it’s really depressing, but I’m at a stage now that yes, I live in constant pain of varying levels, and they help me to find and recognise the joy, and not get too bogged down when everything hurts and I can’t do anything (Sarah, semistructured interview)
6.	Walking [dog] forces me to get outside and do something I might have not done otherwise, and then I usually run into dog walkers and other people and have at least one conversation. Yes, having a conversation is really nice, it helps with not feeling lonely and isolated and stuff, and usually improves my pain by the time I get home (Nicole, semistructured interview).
7.	My support worker is phenomenal. If I didn’t have [support worker]), I wouldn’t have the meals in the freezer that we’d precooked because chopping carrots and everything just hurts. she’s like a little fairy and just does it all, that immensely helps my pain, but also really helps my mental mood. She has changed my life (Sarah, semistructured interview)
8.	[Daniel] played a gig for his friend’s birthday. As more people arrived and were enjoying the music, he felt pretty validated, his girlfriend was incredibly impressed and proud of him, and he enjoyed playing so much he didn’t want it to end. He had no pain at all during the event or in the days following the party (Daniel, field notes)
9.	Horse riding is a bit of an escape, when I get on the horse I forget about the pain. Being on top of the horse makes me feel included in life, because no one knows that I’m in pain, unlike going for a run or swimming, the horse’s legs are my legs. I enjoy it and if I’m in pain when I get off, well it’s worth it (Tara, field note)
10.	She’s enjoying a dance class, as it’s a new way to know her body, it’s for pleasure, it’s playful, it’s collaborative, its socialising, and she talked about how it made her more body aware, more confidence in her body, and a way for her to start to like her body again but also to check-in on the pain (Olivia, field notes)

Some participants found supporting others a helpful distraction from their own pain, though this sometimes led to imbalanced relationships that neglected their own needs and intensified their pain (table 2, quotation 2). Pain was also reported as more severe when social networks were limited or key relationships became strained (table 2, quotation 3). In such moments, feelings of isolation, self-criticism and perceived judgement from others often heightened the experience of pain.

For those with limited social support, pain often worsened when regular contact became difficult, though many found creative ways to stay connected. For instance, pets played a multifaceted role, offering comfort, companionship and a sense of responsibility that influenced how individuals experienced pain (table 2, quotation 4). The routine of caring for their pets provided structure and brief moments of joy, helping to ease the ongoing burden of chronic pain (table 2, quotation 5). One participant described walking her dog during pain flares as a manageable way to stay socially engaged, preferring brief, casual conversations with strangers over more emotionally demanding interactions with close friends (table 2, quotation 6).

In addition to pets, participants valued professional and community-based relationships, often forming meaningful connections with cleaners, support workers and healthcare providers (table 2, quotation 7). Neighbours also emerged as key sources of support, with everyday encounters woven into the rhythms of daily life. Participants described how hobbies also connected them to supportive groups that felt like extended families, offering care beyond the shared activities, such as checking in or helping with errands, especially during periods of heightened pain.

Participants described how creative pursuits and physical activities helped them manage pain and reconnect with life. Daniel found that making music in a band brought joy but also eased his pain, with the positive effects lingering for days (table 2, quotation 8). Daniel reflected on the importance of being open with others, though not always possible, as a way to keep his pain in the background and reduce its impact on life. Similarly, Tara experienced horse riding as a powerful escape, offering a sense of inclusion and freedom. Although the pain often returned afterward, the joy and liberation she felt while riding made it worthwhile (table 2, quotation 9). Another participant described how dance and exercise helped her to develop a new relationship with her body, manage her pain more effectively and live well alongside it (table 2, quotation 10).

Taken together, elements of this theme provide a window onto the value of social connections and how people living in isolated circumstances experience the impact of disruption to social connections in ways that have particular impact on pain and related well-being. The value of all relationships appeared to be related to the degree of safety and associated trust that was felt. This was the case in all forms of relationships, whether close personal connections or those formed around shared interests and activities, such as hobbies.

### The interplay between work, family roles and fluctuations in chronic pain

Participants in paid or voluntary roles described their work as meaningful, offering purpose, structure, social interaction and for some, distraction from pain. At the same time, work contributed to their sense of identity and financial stability. Some people coordinated childcare with their partners so that they were more able to balance family responsibilities, employment and pain. However, when combined household incomes exceeded the threshold for receipt of financial welfare benefits, time for self-care became limited (table 3, quotation 1). A strong sense of obligation to work despite pain was common.

**Table 3:** The interplay between work, family roles and fluctuations in chronic pain

Quotations	
1.	Because we are financially struggling, like not every day, but we do go, oh, my God, and I’m above threshold for [financial social welfare benefits] help because of partner’s wage, my wage, even having three kids it’s not enough. But I can’t be in this limbo where you’ve got to do everything Monday to Friday, all the cleaning, all the cooking, everything and then go to work some evenings and on weekend (Anna, semistructured interview)
2.	Yeah, but then it also means I can… If I’ve got like the option, like if I get offered loads of work then I can choose what I take. So, like at the moment I could probably be working like 7 days a week and whatsit, but I’ll turn things down when I’m in pain and they are a lot more accepting (Daniel, field notes)
3.	Yeah, long days, 10 hours a day for 4 days a week. It’s not that it makes pain worse, but I can feel it more but probably the day off gives me more of a chance to rest. I am exhausted at the end of the week but I’m finding that having an opportunity to rest in the middle of the week has helped [my pain] a bit (Emily, field notes)
4	[Being busy at work] doesn’t make the pain worse - it just means that I might notice the pain more - just a bit like everything can be on a downer, my body feels very heavy because I feel so mentally exhausted. He talked about getting up in the morning and having to drag his legs around (Kevin, field note).
5.	Since lockdown and working from home, employers expected you to be working when you’re ill – whereas that wasn’t the case prior to lockdown because if you were off sick, you were away from your desk ill, but now we have desks at home and more flexible working conditions, so they expect you to still produce work. Today, I found working in the office hard. I did a morning and was in pain and so work colleagues wondered why I’d even come in to work and suggested that I go home, presumably to work from home – not off sick (Logan, field note)
6.	He talked about making new work friends: what I have come to realise is that if I sit here on my days off, on my own and don’t talk to anybody, then I can see how I might end up back in more pain … So, there are couple of older guys at work, and they were chatting about going for a couple pints and a chinwag, so I joined their conversation and asked if they were going again whether they could let me know so that I could join them … I realised, if I have different people in different places that I can talk to, then if something happens to one of them, I still have other outlets (Gabriel, field notes)

To manage pain, some participants turned to ‘zero-hour’ contracts for flexibility (in which their working hours varied considerably), though this option also exposed them to more precarious work and financial instability (table 3, quotation 2). Others preferred shift patterns or compressed hours, which concentrated their work into fewer days by working longer hours each day to allow time for rest and recovery as a way of managing pain (table 3, quotation 3). High workloads and job-related stress were described by participants as intensifying pain. For some, the physical tension of meeting demands intensified their pain; for others, job-related stress did not directly worsen pain but made it more noticeable (table 3, quotation 4) and when work breached its boundaries, opportunities for rest, recovery and self-care were often reduced.

Participants managed pain at work by combining workplace resources, like ergonomic equipment, with personal investment in specialist chairs and standing desks. Flexible, adaptive and supportive workplace policies were viewed as essential for accommodating participants’ pain-related needs, without increasing visibility or discomfort. Working from home was generally viewed positively, offering freedom of movement and pain relief strategies. However, one participant noted that blurred boundaries between work and home sometimes led colleagues to suggest remote working as an alternative to taking sick leave for back pain (table 3, quotation 5).

Participants distinguished between colleagues and work friends, noting differences in the depth of these relationships. Several participants described how relationships with colleagues had grown into meaningful friendships that extended beyond the workplace. One participant reflected that spending days off alone and avoiding social interaction could worsen his pain and that spending time with trusted work friends outside of work provided an important emotional outlet (table 3, quotation 6).

Together, matters relating to work and associated household finances were related to the experience of pain. People living with pain appeared to exert agency where possible and made choices about their work, but financial pressures limited the agency to care for themselves. This push and pull between the benefits and pressures of work was in flux, but agency appeared to be paramount.

### Social spiralling and fluctuations in chronic pain

The analysis identified key moments that influenced changes in people’s experiences of chronic pain. Sometimes these were reported as worsening pain, in a process described by one participant as a ‘spiral’. At other times, social phenomena helped to improve pain. These influences operated across multiple levels (eg, intrapersonal, interpersonal, institutional, community) often overlapping and reinforcing one another.^34 36^ Together they created patterns of social engagement that, in turn, affected how the pain was felt and managed.

For instance, Courtney, a participant with high pain severity and impact, described feeling caught in a downward ‘spiral’ where one difficulty led to another, making it harder to recover (table 4, quotation 1). For her, recognising the onset and existence of the spiral was crucial, as at these times small actions, like getting out of bed or getting dressed, became meaningful wins on days when pain was difficult. Courtney also experienced a series of changes that took place around the same time with one another and that seemed to influence improvement in her pain: she started a college course, expanded her existing work, took on new work and increased her friendship circles (table 4, quotation 2). These changes appeared to work together and amplify one another to support her sense of progress, well-being and positively impact on her pain. Consistent with Courtney’s experiences, Kevin described how his pain was influenced by a series of changes that occurred around a similar time to one another: he started a new job, gained the financial means to settle debts, made home improvements, went on holidays, strengthened relationships and became more socially active (table 4, quotation 3). These interconnected shifts appeared to move together, amplifying the collective impact of social life on his experience of chronic pain.

**Table 4:** Social spiralling and fluctuations in chronic pain

Quotations	
1.	It is like that downward spiral, if one thing starts going wrong, something else, something else. I’ve got to the stage where, if you don’t stop that spiral from happening or acknowledging that you’re going downwards, you can’t get back up … Like for me, when my pain was bad, yes, I’m in bed all day, yes, I’m sleeping, yes, I’m not going to work, I’m not doing this, I’m not doing that. But it might just be something as simple as I get up and go to the toilet, or I get up and get myself some water, get up and get dressed. At least that’s one small positive thing that I can take from that day, that I can think actually, yeah, okay that was really bad day, but I did get up and I did get dressed (Courtney, semistructured interview)
2.	The social side of it has changed because I’m talking a bit more to people and becoming a bit more active and stuff like that … the course has helped, having supportive family and friends have helped, getting a new job has helped … Yes, my back’s in pain, I’ve got to take some tablets, I’m gonna do this (Courtney, semistructured interview)
3.	I don’t know exactly what it is that has made my pain go away, whether it has something to do with different working conditions where I don’t have to stretch so far, whether it’s got something to do with earning more money and being out of debt, going on holiday, getting on well with my partner or being more sociable I’m not sure exactly what it is, but if I do a little bit of everything then I might be able to continue to live without pain (Kevin, field note)
4.	If I had to say what living with pain is like in relation to everyday life, I would say that I am stood on a train platform. Trains are whizzing past in both directions, its noisy and there are lots of people on the platform waiting for the train, the train pulls in on both sides, and everyone gets on the train. As the trains pull away, everything becomes peaceful and quiet again, everyone got on the train, and I look across the platform see pain, stood on the opposite platform, waving at me (Ryan, field note)
5.	I think it’s a mix of your social within your family, you’ve got social aspects in the way of work, you’ve got like just random social experiences in a way of who’d you bump into, like just normal life. You’ve got in-home experiences, you've got personal experiences, you’ve got a lot. It’s everything … Even just walking out to a car, that will impact you potentially (Anna, semistructured interview)
6.	You still know when you’re gonna have a bit of a flare up of some sort. But I think it’s just how you offload these sandbags, because they are heavy. And the more you’ve got on, the heavier that is getting on your back, on your legs, on your spine, on your head. You’re gonna end up with pain if you don’t start offloading them … It’s not just a social aspect; it’s not just a health aspect. It’s everything in one (Anna, semistructured interview)

Another participant, Ryan, described a busy period that included a positive and energising work event, a demanding week at work and a visit from young family members. Ryan enjoyed several outings in the local area with family and the children got along well. Ryan also described reconnecting with a close friend in an unexpected meaningful way at the same time. At this time of rich social engagement, Ryan experienced the most intense pain that he had felt in years, not during the busy period itself but once life began to settle. This counterintuitive pattern, where pain emerged following positive or fulfilling meaningful experiences, may be explained by overexertion as individuals pushed themselves to fully engage in enjoyable activities. This might reflect the classic ‘boom or bust’ cycle, addressed in studies exploring whether pacing is beneficial and in which periods of heightened activity are followed by depletion.^56 57^ Ryan offered a different view: he did not see pain increase after activities as related to exhaustion. Instead, he described how once the distraction of enjoyable interactions had passed and he allowed himself to relax, the pain seemed to return, as if it had been waiting in the background (table 4, quotation 4).

Anna described how the cumulative impact of social phenomena on pain was not necessarily driven by a series of major events, like starting a new job or expanding social circles, but by a constant stream of everyday social and environmental inputs. Her pain was shaped by a wide range of everyday experiences, including family life, work, casual interactions and personal routines (table 4, quotation 5). Even small, seemingly ordinary moments influenced changes in pain, which highlighted how fluctuations are a complex and dynamic part of everyday life. Living with pain while working and caring for both close and extended family sometimes felt overwhelming for Anna. She reflected on how many different pressures and responsibilities were constantly at play (table 4, quotation 6). For her, everything was connected—health and social life were not separate but part of an integrated whole.

The spiral described so clearly by participants indicated that they understood and were able to articulate the complex relationship between pain and layered aspects of everyday life. As in the two previous themes, agency and safety were important and enabled people to exert a degree of control over their lives with pain, where possible. Equally, many activities, pressures and requirements of everyday life were outside the control of individuals, which presented greater difficulty and could exacerbate pain. Taken together, these dynamics highlight how pain was experienced through a constantly shifting interplay between agency, safety and the often-inflexible demands of everyday life.

## Discussion

This ethnographic study examined how individuals navigate the persistent or fluctuating presence of chronic pain within everyday life. Social dynamics, including relationships, everyday routines and occupation, played a central role in shaping how pain was experienced and managed. Analysis indicated that feeling connected and understood was key, that this could be understood through the concept of safety as emotionally supportive relationships were more meaningful than the size of their social networks. Humour was evident as a vital personal resource for managing their chronic pain. Engagement in hobbies and community-based activities fostered connection, distraction and bodily awareness. Employment and volunteering provided purpose and distraction from the pain, though balancing responsibilities could be challenging. Flexible work arrangements supported rest and recovery but sometimes came at the expense of financial stability. In contrast, meaningful workplace friendships offered emotional support and helped to ease the isolation of living with chronic pain.

In this study, all participants described being in a continual process of managing their pain, with fluctuating experiences of both improvement and worsening throughout the year. Taking duration and impact together, Eccleston and colleagues^8^ propose a transitional framework to categorise pain ‘states’, capturing how individuals move between different states (acute to chronic) and varying levels of pain intensity (high or low impact). Transitions between states reflect the changing nature of chronic pain, encompassing worsening, improvement, maintenance and resolution.^8^ Our findings illustrate how fluctuations in chronic pain are shaped by complex, interwoven layers of social connection, from close personal relationships to broader dynamics within communities and workplaces. While pain remained a constant presence within a maintenance state, its intensity and impact (high or low) ebbed and flowed in response to everyday life. This study offers insight into how people manage persistent pain, within this dynamic and socially embedded context.

The longitudinal design of the study reveals how social factors shape chronic pain and how individuals adapt over time, while avoiding reductive causal explanations. Unlike lifestyle-focused research that often implies personal responsibility, this study highlights the complexity of living with pain. Although interventions targeting nutrition and activity are important, they can sometimes be interpreted as conveying a punitive tone in health messaging, implicitly implying that individuals are responsible for their pain due to not making ‘healthier’ choices.^58^,^61^ The need to consider a broader range of modifiable lifestyle factors implicated in chronic pain is further supported by Nijs and colleagues.^61^ Their study, which examined lifestyle and chronic pain across the lifespan, found that current treatment options often fail to fully address the wide range of associated lifestyle factors. This ethnographic research deepens understanding of how multiple, concurrent aspects of lifestyle influence the experience of chronic pain, strengthening the case for more comprehensive, socially informed approaches to care.

Understanding chronic pain requires attention to complex social contexts in which it unfolds, across individual, relational, community and institutional domains.^32^,^3436^ A socioecological perspective supports multidimensional responses that complement medical care, that might include social support, community engagement, lifestyle adaptations and policy change.^38 62^ For instance, Potthorff and colleagues^63^ in a meta-ethnography identify eight social dimensions contributing to the onset and progression of chronic pain, including gender inequity, stigma, marginalisation, harsh living conditions and loneliness. They noted, however, that few qualitative studies explicitly examine social dimensions as explanatory factors. Similarly, Toye and colleagues^64^ synthesised qualitative studies about life with chronic pain, highlighting how journeys of healing are non-linear, deeply personal processes shaped by meaning-making. Responding to calls for deeper exploration of social influences, this ethnographic study offers contextual depth by including different points in individuals’ pain journeys and showing how they manage, conceal, narrate and negotiate pain within their social and material worlds.

A key strength in the study was its longitudinal ethnographic design, which achieved nuanced data about how chronic pain and social dynamics change over time.^39^ The adequacy of the sample was underpinned by achievement of information power, an approach that emphasises the richness and relevance of the data in relation to study aims.^46^ Combined with rigour in analysis, information power supports transferability of the findings to other contexts. The sample’s inclusion via the ALSPAC birth cohort meant that all participants were between 32 and 33 years old. This age range offered an opportunity to examine chronic pain in early midlife, a period often under-represented in pain research.

The study findings align with European evidence about the relationship between pain and work. For example, Fagerlund *et al*,^65^ in a large longitudinal study of early-midlife Finnish municipal employees, reported that recurrent pain was shaped by the interplay between workload demands and a range of health-related factors. Previous qualitative research has highlighted how pain can cause exit from the workforce, for instance in a qualitative study based in the Netherlands.^66^ Relatedly, a body of work focuses on predication and prevention of work absences, with well-established public health and economic reasons for understanding the antecedents of chronic pain.^65 67^ Our study further demonstrates the important relationship between pain and work but provides more detailed information about the ways in which people living with pain were able to exert a degree of agency, for instance through ‘zero hours’ contracts; and how social relationships through work were of value. Conversely, financial pressures exacerbated pain and its impact by reducing time available for self-care.

Furthermore, participants in our study were in their thirties. For these individuals, balancing their own household and financial needs was often paramount, with impact on their pain of which they were well aware. These findings may also inform approaches to the lives and needs of people at later life stages, including consideration of how people might transition well into middle age and then into later life. For instance, UK guidance on assessment of pain in older people already highlights that social isolation can be an indicator or predictor of pain that should be attended to in clinical assessments.^68^ In our study, appreciation of the way in which social relationships dynamically buffer or exacerbate chronic pain at an earlier life stage may be used in the future to inform work across the life course. For example, features of life with pain for people in their thirties may inform design of approaches to prevent onset or reduce persistence of chronic pain as people pass to later life stages. Future research could apply ethnographic, longitudinal methods with older or younger age groups to explore whether social aspects of pain manifest across life stages. That said, a limitation of the study was geographical and demographic diversity: all participants were from one region of England, and all identified as white British. Future research should prioritise inclusion of more diverse and intersectional experiences of chronic pain and consider the potential of the work to deliver benefit to participants and their communities. Achieving this might require community-based approaches that focus on co-produced insights and locally grounded solutions.

## Conclusion

This ethnographic study has identified the complex ways in which chronic pain is embedded within everyday social practice, across multiple and intersecting levels of influence. The study signposts the importance of looking beyond the individual to broader social contexts in the development of interventions that might prevent or reduce the impact of pain. For instance, such an approach invites greater attention to, and investment in, local infrastructure and community-based initiatives. This might include coordinated action across health, social care and community services to address the wider circumstances and living conditions that influence chronic pain.

## Supplementary material

10.1136/bmjopen-2025-111270 online supplemental file 1

## Data Availability

Data are available upon reasonable request.
