# Supplementary material for: Everyday social contexts influence fluctuations into and out of chronic pain: an ethnographic study in England
Source: BMJ Open. 2026 Apr 1;16(4):e111270. doi: 10.1136/bmjopen-2025-111270 (PMC13052564; doi:10.1136/bmjopen-2025-111270)
Supplement: online supplemental file 1 [file bmjopen-16-4-s001.docx]

# Topic Guides

**Authors’ explanatory note: The topic guide was used flexibly across a range of timepoints and topics. It is included here for transparency and completeness. Analysis presented in this article is described in the methods section, which explains focus of analysis that does not necessarily include all of the topic areas or items represented on this topic guide.**

## Interview questions

Semi-structured interview questions used open-ended techniques (e.g., *Can you tell me about how life changed after your chronic pain started?*) and probes (e.g., *Can you tell me more about that?*) to elicit participants’ experiences in their own words. The interviews were designed to be flexible, allowing participants to determine the order in which topics were covered and enabling adaptation to their circumstances. Topic guides were purposely flexible so that unanticipated issues and ideas could emerge, and participants were encouraged to speak freely about matters they considered important.

Interview questions were revised and refined in relation to participants’ social contexts, pain experiences, and emerging themes. Questions were reworded and rephrased in relation to context, and additional questions were added where relevant. Participants were reminded that their participation was voluntary, that they could refuse to answer any question, and that they could end the interview at any time without explanation. The precise phrasing of questions and order of the questions asked was adjusted as necessary during interviews and throughout the study to reflect participants’ language and priorities rather than those of the research team.

## Focus: Lifestyle and pain (Topic 1)

1. Could you tell me a little bit about yourself and your pain condition?
2. Could you tell me how your pain has affected your lifestyle?
3. What was happening when you first started experiencing pain?
4. Is the pain you experience now similar to when the pain started?
5. How do you manage your pain in everyday life?
6. What does your pain look like on a good day and a bad day?
7. What can you do on a good day that you can’t do on a bad day?
8. What do you think influences or trigger your pain on a bad day?

Prompt: sleep, stress, diet, activity, mood

1. How do you manage your symptoms on bad day?

Prompt: go to bed, exercise, call a friend

1. Is there anything you would like to tell me about your chronic pain or lifestyle that I haven’t asked that you think is important for me to know in terms of what makes your pain get better or worse?

## Focus: Friends and social connections (Topic 2)

1. There are no right or wrong answers, can you describe what a friend or friendship looks like for you?
2. What are some of the qualities that you look for in a person to enable you to call them a friend? (in relation to pain or otherwise)
3. Aside from family, do you have someone who you talk to about your pain? (who is your go to person/s?)
4. (And if so) Who are your go to friends if you want to talk about your pain? And Friends you don’t want to talk to about your pain? And why?
5. Do you feel lonely or isolated?
6. How does interacting with your friends affect your pain? (in terms of doing things together)
7. How does your relationship with friends affect your pain? (in terms of friendship over time)
8. Would you say you feel accepted and have a sense of belonging with your friendship group?
9. Have you made new friends though bonding in the pain experience?
10. Do you think your friends understand how your pain impacts your life? (invisibility of pain)
11. How has living with pain affected your social life? (variability of pain)
12. Have you changed the way you socialise? (as a result of living with pain)
13. Do you ever feel you protect your friends from your pain? (do you cover up/downplay your pain in front of them?)
14. Is there anything you would like to tell me about your friends in relation to your pain that I haven’t asked that you think is important for me to know in terms of what makes your pain get better or worse?

## Focus: Resources and managing pain (Topic 3)

1. Can you describe the types of resources you have access to for managing your pain?
   1. Prompt: medical, financial, social, cultural, material, educational, time
2. How do these resources influence your experience of living with pain? (ability to manage and treat your pain)
3. What role does your access healthcare play in the management of your pain? (private or otherwise)
4. How do you use community resources (support groups, local services, etc.) to help manage your pain?
5. Do you think your knowledge or your way of thinking about how medical things work influences your pain? (Knowing about systems of ideas? Language?)
6. Do you think your family and family background are resources? And how do they affect your pain? (question always reworded, often includes work and/or education)
7. Are there any gaps in the resources available to you that make managing your pain more difficult? (question always reworded)
8. What advice would you give to someone seeking to improve their access to resources for pain management?
9. Is there anything you’d like to tell me about your resources in relation to your pain that I haven’t asked that you think is important for me to know in terms of what makes your pain get better or worse?

## Focus: Family and social connections (Topic 4)

1. Could you describe who is in your family? (and how often you see/interact with them)
2. Would you say your family understands the variability of your pain?
3. Do they offer sympathy and concern? (and/or support)
4. Do you ever ask any family members for help due to your pain? E.g., with any day-to-day tasks? With what sort of tasks?
5. How do family members respond to you when you are in pain?
6. In which ways do you feel your family support you when you are in pain?
7. Do you feel living with pain puts strain on your family? (e.g., burden to others)
8. Are there specific family members who are more involved than others?
9. Has your relationship with your family changed since living with pain?
10. Has your pain affected your relationships with extended family? E.g., ability to attend family celebrations or regularly interaction?
11. What advice would you give to other families on supporting a loved one who is in pain?
12. Is there anything you’d like to tell me about your family in relation to your pain that I haven’t asked that you think is important for me to know in terms of what makes your pain get better or worse?

## Focus: Work (Topic 5)

1. Could you describe your work?
2. how does your work relate to you living with pain? (does your work affect your pain)
   1. What are the primary influencers (social, economic reasons etc)
   2. Do you feel comfortable to negotiate different arrangements around your pain? (working conditions/patterns/ flexibility/WFH/appointments/workplace adjustments)
   3. How does working from home relate to your pain condition?
3. Have you ever taken time off work because of your pain?
   1. How do people respond to you if you are unable to work due to your pain condition?
   2. Have you had any issues negotiating the sickness policy?
   3. Is your manager and colleagues understanding of your pain situation?
4. Do you enjoy work and your colleagues?
5. What kinds of support has helped you to stay in work?
6. Is there anything you’d like to tell me about your work context in relation to your pain that I haven’t asked that you think is important for me to know in terms of what makes your pain get better or worse?

## Focus: Parenting with pain (Topic 6)

1. Could you just describe your situation/role in parenting?
2. What are the challenges relating to parenting whilst living with pain?
3. Describe how your pain is influenced by parenting activities?
4. Does your [significant others] understand any challenges you experience in relation to parenting with pain?
   1. If so, how do they support you to parent alongside your pain?
5. Do you ever feel you want/need to hide your pain from family/children?
6. Do you ever feel you need/want to push through the pain, and ignore it in as much as is possible to participate in family activities or social events, accepting that some activities will have future consequences for your pain worsening?
   1. If so, are there any things you do to recover?
7. Is there anything you want to tell me about parenting and living with pain that I haven’t asked that you think is important for me to know in terms of what makes your pain get better or worse?
