# Supplementary material for: Everyday social contexts influence fluctuations into and out of chronic pain: an ethnographic study in England
Source: BMJ Open. 2026 Apr 1;16(4):e111270. doi: 10.1136/bmjopen-2025-111270 (PMC13052564; doi:10.1136/bmjopen-2025-111270)
Supplement: Supplementary file 2 [file bmjopen-2025-111270.reviewer_comments.pdf]

# PEER REVIEW HISTORY

BMJ Open publishes all reviews undertaken for accepted manuscripts. Reviewers are asked to complete a checklist review form and are provided with free text boxes to elaborate on their assessment. These free text comments are reproduced below.

## ARTICLE DETAILS

**Title (Provisional)**

Everyday social contexts influence fluctuations into and out of chronic pain: an ethnographic study in England

**Authors**

Stone, Samantha; Zeyen, Anica; Gooberman-Hill, Rachael

---

## VERSION 1 - REVIEW

---

| | |
|---|---|
| **Reviewer** | **1** |
| **Name** | **Stacey, Stephen** |
| **Affiliation** | **Mayo Clinic** |
| **Date** | **01-Dec-2025** |
| **COI** | **None** |

---

Review: An ethnographic study examining how everyday social contexts influence fluctuations into and out of chronic pain

My review focuses on two primary concerns that warrant substantial revision:

1. The methods section, as currently described, lacks clarity and does not consistently align with the stated analytic paradigm, making it difficult to evaluate the rigor and appropriateness of the approach.

2. The findings remain overly descriptive and do not offer the level of interpretive insight or actionable implications that would allow readers to meaningfully apply the results.

Abstract:

• When reading the abstract alone, it is difficult to discern the specific contributions of the study. For example, statements such as “Fluctuations in chronic pain were complex, shaped by and entangled with social contexts…” and “The findings highlighted how social dynamics played a central role in shaping how pain was experienced” remain fairly broad and do not convey any concrete insights that may have emerged from the analysis. Since it is already well established that chronic pain is complex and influenced by social factors, it would be helpful for the abstract to highlight the particular findings or mechanisms this study

identified. I recommend including more specific results to clarify the study's unique contributions.

Introduction:

• Line 18 has the statistic "just under 28 million people reported to be living with chronic pain in the UK today". My personal preference is to include percentage of individuals since numbers like 28 million are hard to understand without the context of the whole from which the value was derived. This is a minor issue.

Methods:

• The analytic method needs to be described in greater detail. For example, they mention Braun and Clarke's approach (reflexive thematic analysis as the analytic method). I used https://www.thematicanalysis.net/editor-checklist/ to help guide my review. I include a few of the questions it poses:

o Q: Do the authors explain why they are using thematic analysis (TA), even if only briefly? A: No

o Q: Do the authors clearly specify and justify which type of TA they are using? No

o Q: Is the use and justification of the specific type of TA consistent with the research questions or aims? A: No

o Q: Is there a good 'fit' between the theoretical and conceptual underpinnings of the research and the specific type of TA (i.e. is there conceptual coherence)? A: Insufficient data to determine.

o Q: Is there a good 'fit' between the methods of data collection and the specific type of TA? A: Insufficient data to determine.

o Q: Is the specified type of TA consistently enacted throughout the paper? A: No

o There were others that I didn't include here. From what I can tell, the authors' description of their methods does not appear to be consistent with Braun and Clarke's approach. I suggest the authors review the link above, along with the Reflexive Thematic Analysis Reporting Guidelines (RTARG, https://journals.sagepub.com/doi/10.1177/02692163241234800), and use them to guide their reporting of the methods and analysis.

Study Findings

As a clinician who sees patients with chronic pain, I felt the findings remained overly general and did not fully capitalize on the depth of the data. Although the dataset appears rich, the findings remain largely descriptive. Several themes restate common-sense associations (e.g., pain interacts with work and family life) rather than offering analytic insight into the specific processes, mechanisms, or contextual conditions that shape these dynamics. Reflexive thematic analysis typically aims to move beyond surface-level observations toward

conceptual or interpretive contributions. I encourage the authors to deepen their analysis by articulating how these relationships operate, what mediates them, and what new understandings this study contributes for clinicians or researchers working in chronic pain.

Discussion

The authors mention that other age groups have been studied more (last paragraph, page 17). Considering that this study represents a fairly narrow age and demographic range, I suggest the authors compare and contrast their findings with what has been discovered about patients living with chronic pain who are different ages or from other countries/demographic groups.

---

| | |
|---|---|
| **Reviewer** | **2** |
| **Name** | **Ampiah, Paapa Kwesi** |
| **Affiliation** | **Brunel University London** |
| **Date** | **11-Jan-2026** |
| **COI** | **None** |

---

Thank you for the opportunity to review this manuscript. The authors presented a robustly delivered body of work. there are a few things I want to draw their attention to for consideration and amendment. Please see below. I have also identified some spelling mistakes and would recommend a thorough proofread.

Page 2. Abstract. Number 30 - 44

The titles of the themes in your findings do not align with the themes listed in your abstract. Consider listing the them followed by a description. With the way they are written currently, they appear to be statements rather than themes and do not reflect the themes headings in your findings section.

Page 7

Participants

In sampling your participants from the ALSPAC cohort, did you have an exclusion criteria? If yes, please provide details. Also based on your definition for chronic pain to inform your inclusion criteria, did your participants constitute both chronic primary pain and chronic secondary pain participants? You need to make this explicit in your inclusion criteria and provide justification for your choices and how the choices informed participants' responses. I note that you applied the Aches and Pains Questionnaire to recruit your participants. Did you consider the ICD-11 chronic pain classification in determining participant eligibility?

Analysis

Page 9

Number 1 - 20

You analysis section needs to be updated to reflect all aspects of your data collection (I note that you report that you will publish the visual materials later) and how they informed your analysis and results. You need to be clear about how you brought the interview data and observation/field notes data together. From your analysis section, it appears the interviews was the main method that informed your results. If this was the case, you need to be explicit about it. From your findings section, it is clear how you brought these together; however, you need to describe it clearly in your analysis section.

**** The reviewer provided a marked copy with additional comments. Please contact the publisher for full details. ****

---

## VERSION 1 - AUTHOR RESPONSE

| reviewer #1 | | Author response/actions |
|---|---|---|
| 1. | **My review focuses on two primary concerns that warrant substantial revision:**<br>**1. The methods section, as currently described, lacks clarity and does not consistently align with the stated analytic paradigm, making it difficult to evaluate the rigor and appropriateness of the approach.**<br>**2. The findings remain overly descriptive and do not offer the level of interpretive insight or actionable implications that would allow readers to meaningfully apply the results.** | • Thank you for these comments. It is helpful to know that we may improve clarity of the methods and ensure that they enable evaluation of rigour when read alongside the findings. It was also useful to learn that the descriptive nature of ethnographic findings means that there is a need to improve the degree of interpretation and insight, particularly for readers with clinical expertise. To address these comments, we have made some changes throughout the manuscript, that include edits made in response to |

| | | |
|---|---|---|
| | | comments below. These comprise:<br>• Additions to the methods section to improve readability including more introduction to the use of ethnography as a method in health research (page 6), and to the analysis approach used for the fieldnotes as well as the interview material (pages 9-10). The detailed additions relating to analysis are described more fully in our response to comment 4, below. |
| **2.** | **Abstract:**<br>**• When reading the abstract alone, it is difficult to discern the specific contributions of the study. For example, statements such as "Fluctuations in chronic pain were complex, shaped by and entangled with social contexts…" and "The findings highlighted how social dynamics played a central role in shaping how pain was experienced" remain fairly broad and do not convey any concrete insights that may have emerged from the analysis. Since it is already well established that chronic pain is complex and influenced by social factors, it would be helpful for the abstract to highlight the particular findings or mechanisms this study identified. I recommend including more specific results to clarify the study's unique contributions.** | • Thank you, it is useful to know that the abstract needs to describe, with more specificity, the findings of the study. We have amended the abstract so that it explains findings relating to social connections and everyday fluctuations in chronic pain; the interplay between work, family roles and fluctuations in chronic pain; and social spiralling and fluctuations in chronic pain. By drawing together the patterns identified in the first two themes, the third theme contributes |

| | | |
|---|---|---|
| | | important analytical insights that pain fluctuations are produced by interacting social processes that move and shift together (page 1). |
| 3. | **Introduction:**<br>**• Line 18 has the statistic "just under 28 million people reported to be living with chronic pain in the UK today". My personal preference is to include percentage of individuals since numbers like 28 million are hard to understand without the context of the whole from which the value was derived. This is a minor issue.** | • Thank you, we have added a percentage (around 43%) alongside the number (page 4). |
| 4. | **Methods:**<br>**• The analytic method needs to be described in greater detail. For example, they mention Braun and Clarke's approach (reflexive thematic analysis as the analytic method). I used https://www.thematicanalysis.net/editor-checklist/ to help guide my review. I include a few of the questions it poses:**<br>**o Q: Do the authors explain why they are using thematic analysis (TA), even if only briefly? A: No**<br>**o Q: Do the authors clearly specify and justify which type of TA they are using? No**<br>**o Q: Is the use and justification of the specific type of TA consistent with the research questions or aims? A: No**<br>**o Q: Is there a good 'fit' between the theoretical and conceptual underpinnings of the research and the specific type of TA (i.e. is there conceptual coherence)? A: Insufficient data to determine.**<br>**o Q: Is there a good 'fit' between the methods of data collection and the specific type of TA? A: Insufficient data to determine.**<br>**o Q: Is the specified type of TA consistently enacted throughout the paper? A: No**<br>**o There were others that I didn't include here. From what I can tell, the authors' description of their methods does not appear to be consistent with Braun and Clarke's approach. I suggest the authors review the link above, along with the Reflexive Thematic Analysis Reporting Guidelines (RTARG, https://journals.sagepub.com/doi/10.1177/02692163241234800), and use them to guide their reporting of the methods and analysis.** | • We thank the reviewer for letting us know that they have used Braun and Clarke's checklist as well as RTARG to support their evaluation of our description of method. We agree that our approach to reporting method was short, in part this was to retain brevity in the submission, and we agree that readers might value more detail and that doing so enables them to see that we have used TA appropriately. To this end we have been pleased to considerably extend the analysis section, including rationale and a step-by-step description of analysis (pages 9-10). This describes our ethnographic approach and explains why we |

| | | |
|---|---|---|
| | | used thematic analysis to enable flexibility and interpretation of both interview and ethnographic fieldnote-based material. This meant that RTA was a good 'fit' for the work and that there was methodological coherence (as required by RTARG) between the study purpose, assumptions, data generation, analysis and interpretation. Together, RTA was enacted throughout the data collection and analysis, and subsequently throughout the manuscript. We support entirely the aspirations of RTARG and note that our use of reporting tool Standards for Reporting Qualitative Research (SRQR) outlines how the research is reporting in line with current practice standards. We hope that the additions made to the manuscript make our approach and intentions much clearer to readers and we thank the reviewer for suggesting that |

| | | |
|---|---|---|
| | | it is important that we improve our reporting of approach and analysis (pages 9-10). |
| **5.** | **Study Findings**<br>**As a clinician who sees patients with chronic pain, I felt the findings remained overly general and did not fully capitalize on the depth of the data. Although the dataset appears rich, the findings remain largely descriptive. Several themes restate common-sense associations (e.g., pain interacts with work and family life) rather than offering analytic insight into the specific processes, mechanisms, or contextual conditions that shape these dynamics. Reflexive thematic analysis typically aims to move beyond surface-level observations toward conceptual or interpretive contributions. I encourage the authors to deepen their analysis by articulating how these relationships operate, what mediates them, and what new understandings this study contributes for clinicians or researchers working in chronic pain.** | • Thank you. This was a helpful comment about the depth of the analysis and findings. The reviewer is correct that the approaches that we used meant that the dataset is rich. We worked hard to stay true to the data in the presentation of the findings, which can inevitably mean that they appear descriptive. To deepen the findings section, we have added more depth of interpretation, at the end of each section in the findings (pages 13, 15, and 18), which is echoed in revisions that we have made to the conclusion and in parts of the discussion. In relation to the findings, in the first thematic area, which is focused on social connections, we draw out a higher level of interpretation that relates to safety in relationships; in the second thematic |

| | | |
|---|---|---|
| | | area, which is about work and family, we extend the analysis to highlight how participants' agency could be constrained by pressures including the interplay between family and household needs and financial requirements. The third theme illustrates how patterns of social life interact in cascading ways that change, intensify or lessen the experience of pain. In addition, we have made some changes in the discussion to elaborate the relationship between findings and literature in more detail. |
| **6.** | **Discussion**<br>**The authors mention that other age groups have been studied more (last paragraph, page 17). Considering that this study represents a fairly narrow age and demographic range, I suggest the authors compare and contrast their findings with what has been discovered about patients living with chronic pain who are different ages or from other countries/demographic groups.** | • Thank you, we have added more reflection on studies of other age groups, particularly older populations. For instance, we now describe how our study findings align with European evidence showing that persistent pain in early-midlife is shaped by workload demands, health-related factors and in some cases early exit from the |

| | | |
|---|---|---|
| | | workplace. We reflect on how our study findings differ from, or resonate with, these studies, including work with other age groups – notably with older populations. These additions can be found in the discussion (pages 21-22). |
| **Reviewer #2** | | **Author response/actions** |
| **1.** | **Thank you for the opportunity to review this manuscript. The authors presented a robustly delivered body of work. there are a few things I want to draw their attention to for consideration and amendment. Please see below. I have also identified some spelling mistakes and would recommend a thorough proofread.** | • Thank you for your careful review of the manuscript, which we have attended to below. Our apologies that some spelling errors were included. We have worked to correct these in the revised submission. |
| **2.** | **Page 2. Abstract. Number 30 - 44**<br>**The titles of the themes in your findings do not align with the themes listed in your abstract. Consider listing the them followed by a description. With the way they are written currently, they appear to be statements rather than themes and do not reflect the themes headings in your findings section.** | • Thank you, we have amended the abstract to include all of the thematic areas described in the findings (page 1). We have stated more clearly that the third theme flows from the first and second theme. We appreciate this comment, which is in line with comment 2 from Reviewer 1. |
| **3.** | **Page 7**<br>**Participants**<br>**In sampling your participants from the ALSPAC cohort, did you have an exclusion criteria? If yes, please provide details. Also based on your definition for chronic pain to inform your inclusion criteria, did your participants constitute both chronic primary pain and chronic secondary pain participants? You need to make this explicit in your inclusion criteria and provide justification for your** | • Thank you. We have added text to make the selection and exclusion criteria clear to readers. In summary, we identified potential |

| | | |
|---|---|---|
| | **choices and how the choices informed participants' responses. I note that you applied the Aches and Pains Questionnaire to recruit your participants. Did you consider the ICD-11 chronic pain classification in determining participant eligibility?** | participants in ALSPAC based on their responses to two of the items included in the Aches and Pains section of the Life at 30+ questionnaire sent out when participants were approximately 30 years old. These questions were: ‘Have you had any aches or pains that have lasted for a day or longer within the past month?’ and ‘If yes, when did the pain start?’. Potential responses to the question about when the pain started were: ‘Less than 3 months ago’, or ‘More than 3 months ago’. The aches and pains question set repeats several previous pain questions, with some additional items and containing aspects of the Bath Adolescent Pain Questionnaire, the Graded Chronic Pain Scale, and additional questions designed by the CRIISP study team. While the questionnaire does not explicitly refer to ICD-11 criteria to determine chronic pain status, the |

| | | |
|---|---|---|
| | | questions align with the ICD-11 definition of chronic pain (pain that persists or recurs for more than 3 months) and participants in ALSPAC were eligible to take part if they had any form of pain that started 3 or more months ago. This included both chronic primary pain and chronic secondary pain. Furthermore, to reduce any potential burden on individuals who had diagnosis that meant they may be near the end of life and therefore in line with the Declaration of Helsinki, potential participants were excluded if they had a such a diagnosis. We have incorporated these clarifications and added the exclusion criterion to the manuscript (Participants and recruitment section: paragraph 2, pages 6-7). |
| **4.** | **Analysis**<br>**Page 9**<br>**Number 1 - 20**<br>**You analysis section needs to be updated to reflect all aspects of your data collection (I note that you report that you will publish the visual materials later) and how they informed your analysis and results. You need to be clear about how you brought the interview data and observation/field notes data together. From** | • Thank you, it is helpful to know that there was a need to expand the analysis section, which we can now see was unhelpfully short. This |

| | | |
|---|---|---|
| | **your analysis section, it appears the interviews was the main method that informed your results. If this was the case, you need to be explicit about it. From your findings section, it is clear how you brought these together; however, you need to describe it clearly in your analysis section.** | resonates with comment 4 from Reviewer 1. We have expanded the description and provided a step-by-step account of process, added more detail and clarity about how the interviews and ethnographic fieldnotes were brought together as a full dataset in analysis. This was achieved through analytic processes with both the fieldnotes and the interview transcripts, which were considered together as a complete dataset to develop the overall thematic areas (pages 9-10). Also, in data collection processes the observations informed the interviews that were carried out with participants, and vice versa. Within the results section, we include illustrative examples both from notes (fieldnotes) of observations and transcripts of interviews, this comprises ten excerpts from semi-structured interviews and eleven excerpts |

| | | |
|---|---|---|
| | | from fieldnotes. This balance of examples is intended to demonstrate that all data sources were used.<br>• To make it clearer that the study, including analysis made use of interviews and observational fieldnotes have made additions both to the data collection section (pages 8-9) and to the analysis section (pages 9-10). |
| **Reviewer #2 In-text comments** | | **Author response/actions** |
| **5.** | **Abstract line 22**<br>**I think the word chronic is missing before pain here.** | • Thank you, we have added the word 'chronic' (page 1). |
| **6.** | **The titles of the themes in your findings do not align with the themes listed in your abstract. Consider listing the them followed by a description. With the way they are written currently, they appear to be statements rather than themes and do not reflect the themes headings in your findings section.** | • Thank you for noting this, please accept our apologies. As described in response to reviewer's comment 2 (above), we have edited the abstract to ensure that the theme headings in the findings section are accurate. |
| **7.** | **Check spelling [differences]** | • Thank you, we have checked and amended spelling errors. |
| **8.** | **Page 7**<br>**Participants**<br>**In sampling your participants from the ALSPAC cohort, did you have an exclusion criteria? If yes, please provide details. Also based on your definition for chronic pain to inform your inclusion criteria, did your participants constitute both chronic primary pain and chronic secondary pain participants? You need to make this** | • Thank you. We were grateful for this comment and have responded in full above to reviewer's comment 3 (Participants and |

| | | |
|---|---|---|
| | **explicit in your inclusion criteria and provide justification for your choices and how the choices informed participants' responses. I note that you applied the Aches and Pains Questionnaire to recruit your participants. Did you consider the ICD-11 chronic pain classification in determining participant eligibility?** | recruitment section: paragraph 2, pages 6-7). |
| **9.** | **Page 6**<br>**Delete [were]** | • Thank you, we have deleted the word 'were'. |
| **10.** | **Analysis**<br>**Page 9**<br>**Number 1 - 20**<br>**You analysis section needs to be updated to reflect all aspects of your data collection (I note that you report that you will publish the visual materials later) and how they informed your analysis and results. You need to be clear about how you brought the interview data and observation/field notes data together. From your analysis section, it appears the interviews was the main method that informed your results. If this was the case, you need to be explicit about it. From your findings section, it is clear how you brought these together; however, you need to describe it clearly in your analysis section.** | • Thank you, it was helpful to know that there was a need to expand the analysis section. Our response is described in full in response to reviewer's comment 4. |

## VERSION 2 - REVIEW

| | |
|---|---|
| **Reviewer** | **2** |
| **Name** | **Ampiah, Paapa Kwesi** |
| **Affiliation** | **Brunel University London** |
| **Date** | **04-Mar-2026** |
| **COI** | |

Thank you for the opportunity to review this manuscript again. I can confirm that all comments that I raised previously have been addressed sufficiently and I am happy for the manuscript to be published.
